# *Elizabethkingia miricola* Causes Intracranial Infection: A Case Study

**DOI:** 10.3389/fmed.2021.761924

**Published:** 2021-12-24

**Authors:** Hongguang Gao, Tian Li, Li Feng, Shu Zhang

**Affiliations:** ^1^Department of Emergency Medicine, West China Hospital, Sichuan University, Chengdu, China; ^2^School of Basic Medicine, Fourth Military Medical University, Xi'an, China

**Keywords:** *Elizabethkingia miricola*, intracranial infection, case report, neurology, bacterial infection

## Abstract

**Background:** *Elizabethkingia miricola* is a rarely encountered bacterium in clinical practice. It is a rare gram-negative rod-shaped bacterium associated with lung and urinary tract infections, but never found in cerebrospinal fluid. This paper reports a case of an adult patient infected by *E. miricola* via an unknown route of infection causing a severe intracranial infection. *Elizabethkingia miricola* was detected by culture and Metagenomic next generation sequencing in CSF. Early identification of this strain and treatment with sensitive antibiotics is necessary to reduce morbidity and mortality.

**Case Report:** A 24-year-old male was admitted to a West China Hospital because of headache and vomiting for 2 months. Symptom features included acute onset and long duration of illness. Notably, headache and vomiting were the primary neurological symptoms. Routine cerebrospinal fluid culture failed to identify the bacterium; however, *Elizabethkingia miricola* bacterium was detected via second-generation sequencing techniques. *Elizabethkingia miricola* was found to be a multi-drug resistant organism, hence, treatment with ceftriaxone, a commonly used drug for intracranial infections was ineffective. This strain eventually caused severe intracranial infection resulting in the death of the patient.

**Conclusion:** In summary, this study comprehensively describes a case of an adult patient infected by *E. miricola* and discusses its early identification as well as application of sensitive antibiotics in the emergency setting.

## Introduction

*Elizabethkingia miricola* is an aerobic, non-budding, non-fermenting, non-motile, elongated, and slightly curved Gram-negative bacillus that causes bacteremia in humans (1). The first case of human disease caused by this bacterium was identified in the United States in 2008 (2). *E. miricola* is a pathogenic bacterium, with reports of bacteremia resulting in sepsis and pulmonary abscesses (3). At present, there are few cases of infection caused by *Elizabethkingia miricola* found clinically. There were about eight cases reported in the literature (3). Most of the pathogenic bacteria in these cases were detected from blood or sputum and caused pulmonary infection or bacteremia, 1 case caused urinary tract infection (4) and 1 case caused oral infection (5). Based on extensive research, only a few cases of adult intracranial infection caused by *E. miricola* have been reported. Herein, for the first time, we identified a case in clinical practice, where *E. miricola* in cerebrospinal fluid and tissue was detected through metagenomic next generation sequencing (mNGS).

## Case Presentation

**Figure 1:**
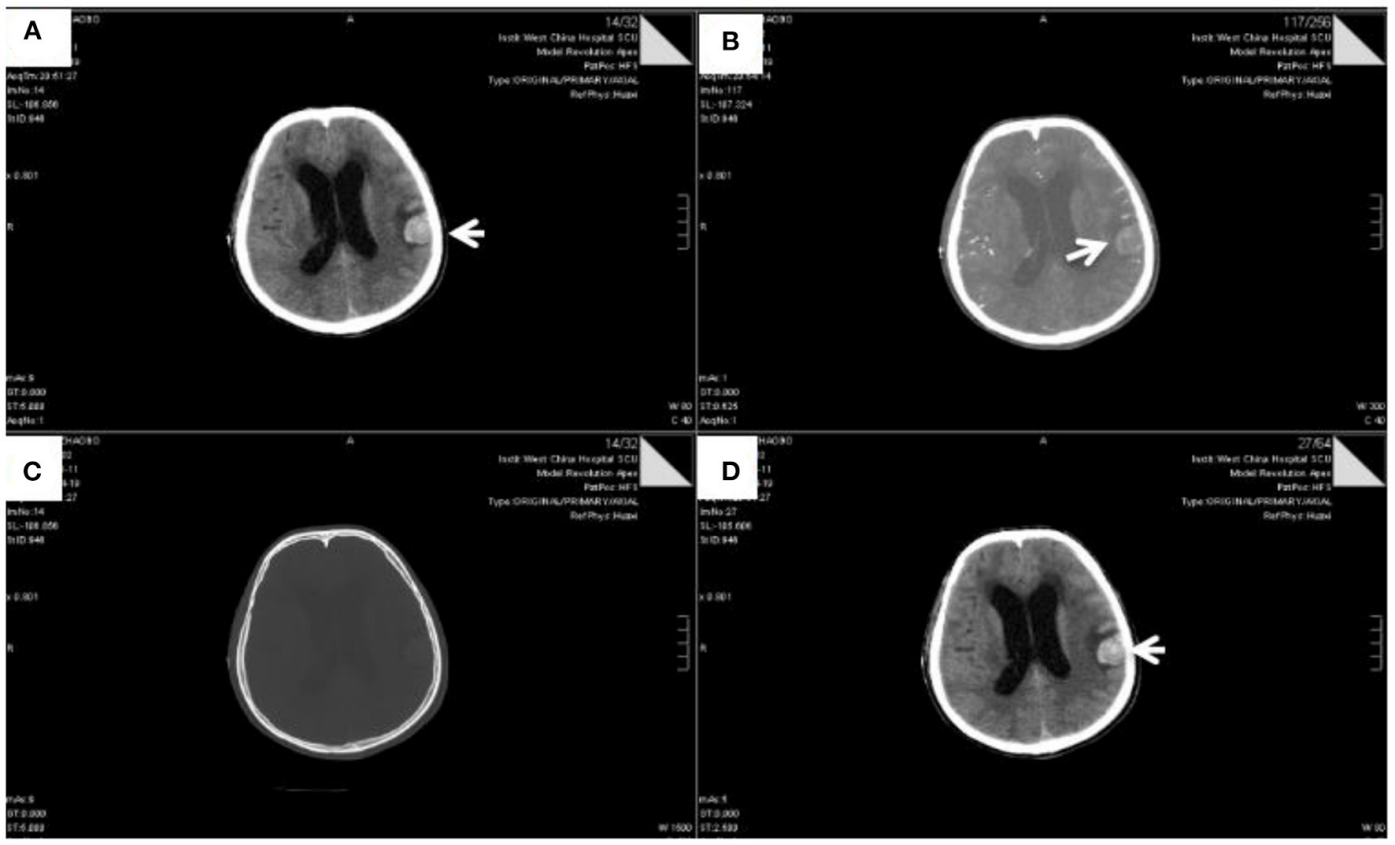
The head CTA scan of patient (Apr 19, 2021). Slightly high-density nodules were observed in the left frontal lobe and temporal lobe. The largest nodules were located in the frontal lobe, with a size of about 2.8 × 2.4 cm (Arrow indicating part). Shadow shuttling of arterial vessels was observed at the edge, without obvious thickening and tortuosity. The intraventricular system was hydrocephalic and dilated, accompanied by interstitial cerebral edema. Some sulci in bilateral cerebral hemispheres became narrow and unclear, and the density of tentorium cerebellum increased slightly.

**Figure 2:**
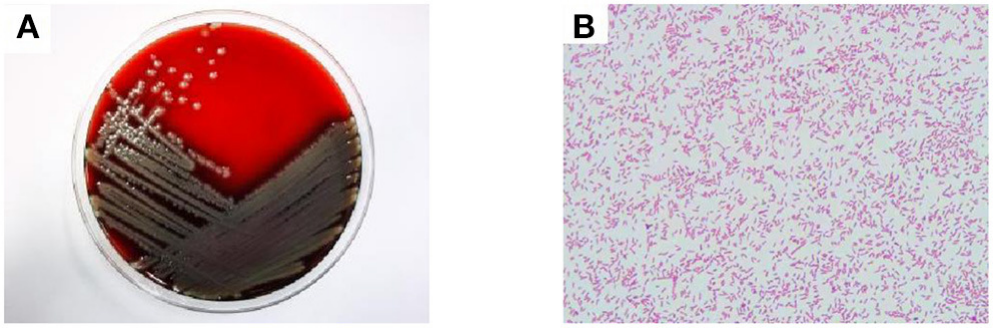
Culture plates showing the growth of *Elizabethkingia miricola*. The blood plate colony is yellow, translucent, round, with neat edges **(A)**. Gram-negative bacilli can be seen 1,000 times under the oil lens of blood culture **(B)**.

**Figure 3:**
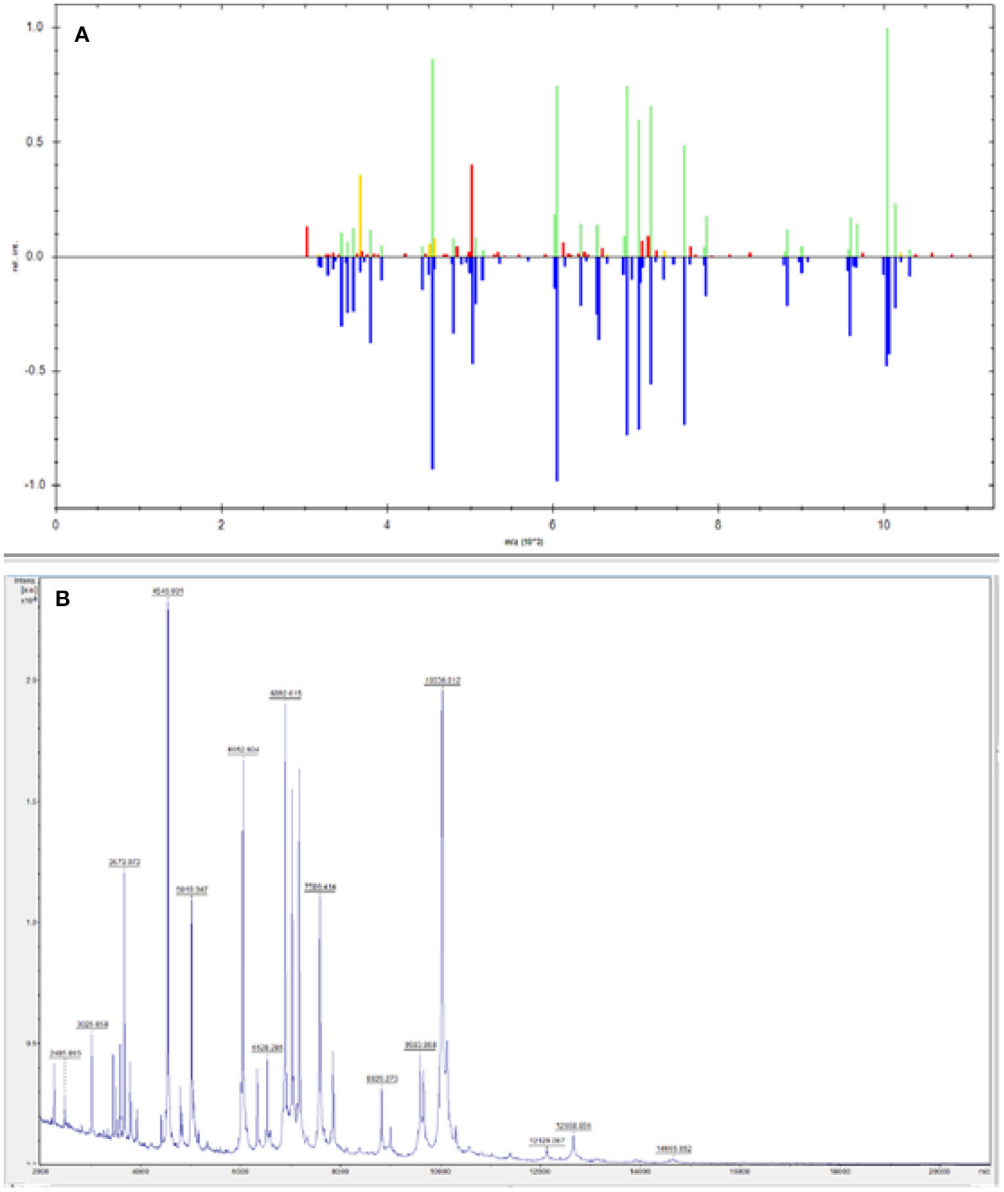
The 16s sequencing comparison result of blood culture samples from patients was Elizabethkingia, and the final sequencing result was *Elizabethkingia miricola* **(A)**. NCBI comparison of 16S sequencing results **(B)**. Primer: 27F AGTTTGATCMTGGCTCAG; Reverse: 1492R GGTTACCTTGTTACGACTT. The 16S amplified sequence of the sample was 100% compared with that of *Elizabethkingia miricola*.

A 24-year-old male patient was admitted to West China Hospital, with “headache and vomiting for 2 months, aggravated for 1 day.” The patient developed dizziness and headache with nausea, vomiting of gastric contents, and coughing out white sputum after a cold during the past 2 months. In the other hospital, the examination revealed “left frontotemporal mass with peripheral edema zone, ventricular fluid, and interstitial cerebral edema.” In the previous month, the patient developed intermittent mental and behavioral abnormalities, with occasionally visual and auditory hallucinations. Lumbar cistern drainage was placed in the local hospital. Then, in the past 5 days, the patient lost consciousness with tonic convulsions of the limbs, double eye gaze, and dental closure. These symptoms lasted for about 1 min, then the patient gradually regained consciousness 10 h later. Subsequently, the patient was transferred to West China Hospital. Admission examination revealed T36.0°C, Pulse 90 times/min, Respiratory rate 20 times/min, Blood pressure 148/108 mmHg, SPO_2_ 97%; drowsiness, accurate and conscious, skin flakes on both lower limbs in front of the tibia; both pupils were equal in size and round. There are no abnormalities in light reflex, heart sounds, respiration sounds and rate. Moreover, the whole abdomen was soft, without pressure pain or rebound pain. A hard node was palpated on the left buttock. The muscle strength of limbs was grade IV. Bilateral pathological signs were negative; the neck resistance was positive, and no edema was found in both lower limbs. The nucleic acid test for COVID-19 of the patient was negative on the day of admission. Computed tomography angiography (CTA) of the head showed slightly dense nodules in the left frontal and temporal lobes, the larger of which was located in the frontal lobe with a size of about 2.8^*^2.4 cm, surrounded by a hypodense edematous band. The ventricular system was filled with fluid and dilated with interstitial cerebral edema. Moreover, extensive soft tissue swelling in the abdominopelvic cavity, subcutaneous fluid accumulation, a nodular and lamellar hypodense shadow in the left gluteus maximus and gluteus medius muscles were observed, suggesting a tendency of inflammatory lesions (Figure 1). According to cerebrospinal fluid examination, cerebrospinal fluid routine showed nucleated cells 16^*^106/L. Secondly, cerebrospinal fluid biochemistry showed 0.34 mmol/L of glucose; 1.85 g/L of microprotein; 105 mmol/L of chlorine. Besides, cerebrospinal fluid bacterial culture identified *Elizabethkingia meningoseptica* (++++) (Figure 2). Drug sensitivity results showed resistance to multiple antibiotics (Table 1) (6). All immunity tests, parasites, and viruses were shown negative. The GM test and G test were negative. CSF ink staining was negative. Candida was isolated in sputum and cerebrospinal fluid culture on the 10th day. The third day of admission, cerebrospinal fluid mNGS results identified *Elizabethkingia miricola* and *Candida*. The 10th day of admission, ultrasound-guided puncture tissue from a gluteal node was subjected to mNGS and *Elizabethkingia miricola* was identified. The cerebrospinal fluid culture specimen was subjected to PCR and mass spectrometry analysis, where it was also identified as *Elizabethkingia miricola* (Figure 3). According to the clinical symptoms and signs, combined with the results of cerebrospinal fluid culture, the patient was diagnosed as bacterial meningoencephalitis (7).

**Table 1:** Cerebrospinal fluid culture and drug sensitivity.

**Identification results: meningeal septic Elizabeth gold bacteria Colony count: much (++++)**				
**Drug susceptibility results:**				
**Antibiotic**	**Results**	**Sensitivity**	**Methods**	**Breakpoints**
Minocycline	28	Resistance	KB	–
Ticarcillin	≥128	Resistance	MIC	–
Ticarcillin/clavulanic acid	≥128	Resistance	MIC	≤ 16, ≥12
Ceftizoxime	≥64	Resistance	MIC	≤ 8, ≥64
Ceftazidime	≥64	Resistance	MIC	≤ 8, ≥32
Cefepime	≥64	Resistance	MIC	≤ 8, ≥32
Meropenem	≥16	Resistance	MIC	≤ 4, ≥16
Amikacin	≥64	Resistance	MIC	≤ 16, ≥64
Ciprofloxacin	0.5	sensitivity	MIC	≤ 1, ≥4
Compound sulfamethoxazole	≤ 1/19	sensitivity	MIC	≤ 2, ≥4
Tigecycline	2	sensitivity	MIC	–
Cefoperazone/sulbactam	22	sensitivity	KB	≥21, ≤ 15
Piperacillin/tazobactam	≥128	Resistance	MIC	≤ 16, ≥128
Cefazolin	≥64	Resistance	MIC	–
Cefatriaxone	≥64	Resistance	MIC	≤ 8, ≥64
Cefotaxime	≥64	Resistance	MIC	≤ 8, ≥64
Aztreonam	≥64	Resistance	MIC	≤ 8, ≥32
Imipenem	≥16	Resistance	MIC	≤ 4, ≥16
Tobramycin	≥16	Resistance	MIC	≤ 4, ≥16
Levofloxacin	0.6	sensitivity	MIC	≤ 2, ≥8
Tetracycline	≥16	Resistance	MIC	≤ 4, ≥16
Piperacillin	≥128	Resistance	MIC	≤ 16, ≥128

*MIC, minimum inhibition concentration, indicating the lowest antibiotic concentration that can inhibit bacterial growth in vitro (μg/ml)*.

*K-B method, disk diffusion method*.

*With reference to the 2014 CLSI document standards, drug sensitivity results were reported according to CLSI M39-A4 (3)*.

## Treatment History

The patient was admitted and then started treatment with ceftriaxone for 3 days, acyclovir and mannitol dehydration (Table 2). The patient was dehydrated with mannitol and glycerin fructose throughout the treatment period to reduce intracranial pressure. The antibiotic was later substituted by cefoperazone sulbactam and on next day the patient was referred to the neurology department for further treatment. After admission, treatment with mannitol and glycerol fructose dehydration was continued to reduce intracranial pressure. After consultations with the infection department, moxifloxacin was added to the treatment regimen immediately, which was changed into cefoperazone sulbactam. Later, after getting yeast-like fungi from culture of cerebrospinal fluid, then an antifungal, voriconazole was added into the treatment. During treatment period, the mental disorder of the patient continued to aggravate, with irrelevant answers, wrong character orientation, as well as progressively worse time and place orientation. The patient became unconscious, and peripheral oxygen saturation dropped to 77%, thus an urgent tracheal intubation was performed with ventilator-assisted breathing. The blood pressure dropped to 56/37 mmHg and the patient was treated with rehydration and dobutamine. The patient continued to be unconscious and his pupil reflex to light was blunted. The family of the patient refused to continue with the treatment and signed out of the hospital. After leaving the hospital, the death of the patient was later confirmed via telephone follow-up.

**Table 2:**
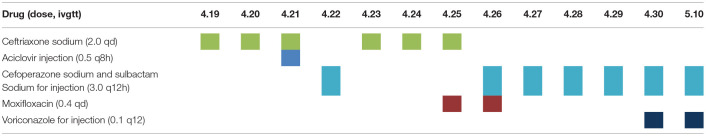
Medication used by the patient.

*The color marks aimed for better distinguishing only; 5.10 means on May 10, 2021*.

## Discussion

This is the first report of a case in which *Elizabethkingia miricola* was detected in cerebrospinal fluid. At present, *Elizabethkingia miricola, Elizabethkingia meningosepticum*, and *E. anophelis* all belong to the Elizabethkingia. It is clinically pathogenic. There were five different lactamase coding genes and 18 efflux protein coding genes in the resistance. Forty-four genes encoding virulence factors were conserved in the strain. Sialic acid transporter and capretin synthesis genes were well-preserved in *E. meningosepticum* but absent in *E. anophelis* and *E. miricola* (3). Numerous clinical reports on *Elizabethkingia meningosepticum*, a Gram-negative bacillus (G–) without pods and budding cells have been documented; this bacterium is one of the conditional causative agents of nosocomial infections. The patient's initial CSF culture was *E. meningosepticum*. Microscopically, the distinction between *E. meningosepticum* and *E. miricola* is limited. However, the metagenomic next generation sequencing (mNGS) result of cerebrospinal fluid was *Elizabethkingia miricola*, so the laboratory conducted further PCR amplification test, and the result was *Elizabethkingia miricola*. *E. meningosepticum* is a multi-drug resistant bacterium, neonates and preterm infants are highly susceptible to it, which results in septic meningitis and sepsis (8). The bacterium is affecting the old or immunocompromised people; patients with multiple severe underlying diseases, or those treated with many broad-spectrum antibacterial drugs; patients with a prolonged stay in the ICU, or under invasive treatment (9). *Elizabethkingia anophelis*, initially isolated from the midgut of the mosquito malaria vector Anopheles gambiae (10), has also been associated with similar severe infections (pneumonia, catheter-related infection and central nervous system infections) with high mortality rates (11, 12). *E*. *miricola* was isolated for the first time in 2003 from the condensation water in the space station Mir. The first case of *E. miricola* (pneumonia and sepsis) infection was reported in 2008, the bacterium was detected from a male patient with lymphoma and under mechanical ventilator support (2). In 2015, one case of bacteremia caused by *E. miricola* in a patient with acute alcoholic pancreatitis was reported in Italy (13). Martina Colapietro identified a novel BlaB variant (BlaB-15) from *E. miricola* isolated from a child with a complicated clinical condition (14). Notably, a degree of immunocompromise is a common feature in these cases, and the long-term oral corticosteroids required for treatment of allergic bronchopulmonary aspergillosis in patient may have predisposed the patient to *E. miricola* infection. However, *E. miricola* was recently identified to cause UTI in an immunocompetent adult (4). The patient, in this case, was a young male with no previous immune-related or other illnesses and no history of trauma or surgery. Therefore, the route of *E. miricola* infection was unknown.

For the first time, *Eligabethkingia miricola* was isolated from cerebrospinal culture. Analysis through conventional cerebrospinal fluid culture identified *Elizabethkingia meningosepticum*. Consequently, it was confirmed that the actual pathogenic organism infected in this patient was *Elizabethkingia miricola*. With the current advancements in detection methods, mNGS is a widely used technique in the diagnosis of early infectious diseases in emergency medicine; providing a more accurate pathogenic microbiological basis for rapid and accurate treatment of patients with infectious diseases.

In the present case, the cerebrospinal fluid culture showed that *Elizabethkingia miricola* was sensitive to ciprofloxacin, levofloxacin, cotrimoxazole, tigecycline, cefoperazone/sulbactam, but resistant to ceftriaxone and imipenem, etc. The imipenem/imipenem-EDTA Etest (bioMérieux, Marcy l'Etoile, France) for detection of metallo-β-lactamase was positive. Although *Elizabethkingia miricola* is a multidrug-resistant bacterium, different studies have reported inconsistent drug sensitivity. Previous studies have shown that *Elizabethkingia miricola* is resistant to ampicillin, ceftazidime, gentamicin, imipenem, cotrimoxazole, macromycin, but susceptible to vancomycin, ciprofloxacin, and rifampin to different degrees (2, 15). Parakriti Gupta (4) reported that *Elizabethkingia miricola* is resistant to both ciprofloxacin and levofloxacin. Generally, limited clinical reports, inconsistent drug sensitivity profiles, lack of antimicrobial susceptibility breakpoints, and a clear consensus on empirical treatment strategies complicate the treatment of these rare microorganisms. As such, early culture of CSF to identify the pathogenic microorganisms and performing their antimicrobial susceptibility testing will be helpful in emergency treatment.

*Elizabethkingia miricola* was detected in cerebrospinal fluid for the first time. This will provide basis for the future research and we can better understand the bacteria. At the same time, the mNGS method was used in this patient to detect pathogenic microorganisms more accurately, providing reference for early clinical diagnosis and treatment of infected patients.

## Data Availability Statement

The original contributions presented in the study are included in the article/supplementary material, further inquiries can be directed to the corresponding author/s.

## Ethics Statement

The studies involving human participants were reviewed and approved by Ethical committee of West China Hospital, Sichuan University. The patients/participants provided their written informed consent to participate in this study. Written informed consent was obtained from the individual(s) for the publication of any potentially identifiable images or data included in this article.

## Author Contributions

HG: writing—original draft preparation. TL and LF: writing-review and editing. SZ: idea. All authors read and approved the final manuscript.

## Funding

This work was funded by 1·3·5 project for disciplines of excellence, West China Hospital, and Sichuan University (ZYJC21055).

## Conflict of Interest

The authors declare that the research was conducted in the absence of any commercial or financial relationships that could be construed as a potential conflict of interest.

## Publisher's Note

All claims expressed in this article are solely those of the authors and do not necessarily represent those of their affiliated organizations, or those of the publisher, the editors and the reviewers. Any product that may be evaluated in this article, or claim that may be made by its manufacturer, is not guaranteed or endorsed by the publisher.
